# Optical Sensing Properties of New Innovative Materials: Interaction of Photoactive Copolymers with Fluorescent Nanoparticles to Create Light-Sensitive Hydrogel Films

**DOI:** 10.3390/gels12030202

**Published:** 2026-02-28

**Authors:** Oscar G. Marambio, Tomás Valdés, Héctor Díaz, Rudy Martin-Trasancos, Julio Sánchez, Guadalupe del C. Pizarro

**Affiliations:** 1 Departamento de Química, Facultad de Ciencias Naturales, Matemáticas y Medio Ambiente, Universidad Tecnológica Metropolitana (UTEM), J. P. Alessandri 1242, Santiago 7800002, Chile; tvaldes@utem.cl (T.V.); hdiaz@utem.cl (H.D.); 2 Departamento de Química de los Materiales, Facultad de Química y Biología, Universidad de Santiago de Chile (USACH), Avenida Libertador Bernardo O’Higgins n 3363, Estación Central, Santiago 9170002, Chile; rudy.martin@usach.cl; 3 Departamento de Química Orgánica, Facultad de Química y de Farmacia, Pontificia Universidad Católica de Chile, Santiago 7820436, Chile; julio.sanchez@uc.cl

**Keywords:** optical sensing properties, light-sensitive films, morphological surface, spiropyrans

## Abstract

This work investigates the use of two photoactive polymers, functionalized with quantum dots (QDs) (ZnS and CdTe/ZnS), to develop optical sensing hydrogel films through their interactions. It examines their responses to light stimulation for potential biological applications. The optical and morphological properties of the films were studied, revealing photoactive surfaces. The photoactive copolymers were synthesized based on poly(maleic anhydride-alt-2-methyl-2-butene), P(MAn-alt-2MB), and poly(maleic anhydride-alt-1-octadecene), P(MAn-alt-OD), by attaching the photochromic agent, 1-(2-hydroxyethyl)-3,3-dimethylindoline-6-nitrobenzo pyran (***SP***). Subsequently, QD nanoparticles (ZnS or CdTe/ZnS NPs) were incorporated into the polymer solutions in the presence of a crosslinker agent, and were then spin-coated onto glass substrates under suitable conditions to produce porous-patterned films. These films were created using a one-step bio-inspired process called the breath figure (BF) method. SEM images of QD-containing samples show a photoactive porous surface resulting from a synergistic interaction between the components. The reversibility of these macroscopic properties results from photoinduced transformations at the molecular level. The light-emitting properties of the films were characterized by blue and violet fluorescence under UV light. Advances in film-forming techniques enable the creation of functional structures with important applications, such as microstructured hydrogel films for biological uses.

## 1. Introduction

Fluorescence tagging of biological molecules, used in the development of immunoassays, cellular labeling, and tissue imaging, is a standard technique in biology; it mainly relies on traditional organic fluorophores and fluorescent proteins [1,2]. In this context, spiropyran is a versatile tool for biomedical applications, ranging from detection and diagnosis to targeted therapy, owing to its responsiveness to external stimuli and tunable functionality. Spiropyran can be turned on and off by light, enabling control over its interaction with target metal ions [3]. Recently, spiropyran-based materials, which can be activated by low-energy near-IR (NIR) two-photon light irradiation and up-conversion nanoparticles, have attracted significant research interest and are reviewed as a new perspective for advanced applications [1]. These properties make spiropyran a versatile and effective tool for scientific and technological purposes, especially in molecular detection and analysis. In this context, spiropyran is utilized as a foundational material for chemical and biological sensors due to its ability to detect a wide range of organic and inorganic molecules [4].

Spiropyran exists in two forms: a closed spiropyran (***SP***) and an open merocyanine (**MC**). The transition between these forms can be initiated by UV and visible light. Spiropyran and its derivatives are versatile photochromic compounds used to detect various molecules and ions because they can switch between the resonance forms of the open merocyanine **(MC**) isoform. The **MC** form has multiple resonance structures, which exhibit different optical spectra depending on the environment [5]. External factors, including metal ions, anions, acids, solvents, and biomolecules, influence the reversible changes in absorbance or fluorescence spectra of the **MC** isoform. These changes enable spiropyran to act as a sensor for detecting these species [6,7]. Spiropyran derivatives with ion-binding sites can form complexes with metal ions, stabilizing the polar **MC** form. This interaction is monitored by changes in optical spectra, enabling colorimetric or fluorometric detection. For example, spiropyran derivatives have been used to detect Zn^2+^, Cu^2+^, and Hg^2+^ ions with high sensitivity and selectivity [8,9,10]. They can also detect anions such as cyanide (CN^−^) and pyrophosphate (P_2_O_7_^4−^) through specific interactions that modify the resonance forms of **MC**. For instance, a spiropyran functionalized with zinc porphyrin changes color upon binding with CN^−^ ions [11,12]. The **MC** isoform is considerably more basic than **SP**, and its protonation shifts in the optical spectra. This property has been used to develop acid vapor sensors and pH-responsive materials. Additionally, spiropyran exhibits solvatochromic properties, making it useful for detecting changes in solvent polarity [13]. This interaction is used to detect and measure nucleic acids. Spiropyran has also been used to detect biomolecules, such as glutathione (GSH) and horseradish peroxidase (HRP) [14].

On the other hand, luminescent semiconductor nanocrystals, such as binary CdSe and CdTe nanocrystals or metal-doped AgInS_2_- and CuInS_2_-core QDs, offer several unique properties and hold great promise for various imaging and bioanalytical applications [15,16]. Additionally, a group of QDs exhibits a broad absorption spectrum spanning the UV to the band edge, allowing for different QD sets emitting at separate optical windows to be excited with a single wavelength. This trait makes them ideal for multiplexing applications. Since early reports emphasized the potential of QDs in biology, there has been a rise in bio-inspired demonstrations, including the detection of soluble substances and imaging of live cells and tissues [17]. However, successfully integrating QDs into biotechnology will require a thorough understanding of these hybrid systems.

Water-soluble quantum dots are becoming more important as tools for biological imaging and sensing because of their unique photophysical properties. Quantum dots are semiconductor nanoparticles made of cadmium selenide (CdSe) or cadmium telluride (CdTe) with a zinc sulfide (ZnS) shell. Their outstanding spectroscopic properties have enabled new applications in the biomedical field. However, improving the solubility of quantum dots requires effective, chemoselective bioconjugation methods. Semiconductor nanoparticles, often called quantum dots, have distinctive optoelectronic properties that depend on their size and shape. Recently, these properties have gained significant interest in the biomedical field, supporting applications such as real-time tissue imaging (bioimaging), diagnostics, single-molecule probes, and drug delivery, among others. The optical properties of quantum dots can be adjusted by their size and composition. Their high brightness, resistance to photobleaching, multiplexing capability, and high surface-to-volume ratio make them ideal for intracellular tracking, diagnostics, in vivo imaging, and targeted therapy delivery [18].

In the biomedical field, QDs provide many benefits over traditional organic dyes and fluorescent compounds [19]. However, certain factors must be considered when these materials are used in contact with biological environments, such as water solubility, stability, and toxicity.

In contrast, quantum dots have garnered substantial interest in biomedical imaging over the past few decades. They are extensively researched for various applications, including DNA-functionalized probes for detecting disease-related biomolecules, diagnostic agents for tumor identification, and molecular probes for simultaneous tracking and monitoring of drug delivery and therapeutic effectiveness [20,21]. Additionally, inorganic materials are easier to process and more resistant to degradation under physiological conditions compared to organic materials [22].

At the same time, nanometer-sized inorganic particles are particularly promising in biomedicine due to their tunability and durability [22,23]. Their nanoscale size increases the surface area-to-volume ratio and enables surface modification to enhance biocompatibility, solubility, and reactivity [24]. In bioimaging, nanoscale particles offer a significant advantage by minimizing the severe toxicity commonly associated with inorganic materials. Their smaller size enables increased sensitivity and allows for a lower effective dose while maintaining practical functionality. Various types of inorganic nanoparticles have been developed for this purpose, including superparamagnetic, metallic, and semiconductor nanoparticles [24].

In recent years, significant progress has been made in photoswitchable spiropyran-based polymers, driven by the unique physicochemical properties of their isomers, which, under various stimuli, including intense light, can activate them. Light is an intriguing and eco-friendly stimulus due to its remote-control ability, its focusable micron- or submicron-sized area with adjustable wavelength and energy, its non-invasive and non-destructive nature, its precisely controllable direction, and its abundance.

Stimuli-responsive materials, also known as innovative materials, are advanced, high-performance substances that respond to external stimuli such as light, temperature, pressure, mechanical force, humidity, pH, and electrical or magnetic fields. These materials undergo reversible changes in their chemical and physical properties in response to these stimuli [25,26,27]. Innovative hydrogel materials have attracted significant attention across diverse fields, including sensors, self-healing coatings, shape-memory and thermochromic materials, optical devices, security documents, and bio-based systems [15,16,17,18,19,20,21,22,23]. In recent years, these photoresponsive materials have been widely used in smart devices that enable controlled physical or chemical changes [24,28,29,30,31,32,33,34]. They have garnered significant attention for their exceptional capabilities in reversible optical data storage [35], photoswitchable fluorescence and cell detection [36], coating [5], ion sensors [37], nanotechnology, and nanomedicine [38,39,40]. The reversibility of these macroscopic properties results from photoinduced transformations at the molecular level [41,42]. These processes can adjust optical signals, enabling the design of optical devices that utilize molecular components [43]. The alteration in chemical structure allows for absorption in a specific spectral region, typically within the visible range. Following a second radiation or thermal stimulus, it returns to its original state [44,45]. Additionally, changes in the absorbance or fluorescence spectra of the material are also reversible and can be influenced by environmental factors [46]. Additionally, the photoresponsivity function must not interfere with monomer polymerization.

These applications include photocontrolled actuation, reversible changes in viscosity, wettability, and polarity, as well as biological applications and photoactivated optochemical responses [47].

On the other hand, quantum dots are semiconductor nanoparticles made of cadmium selenide (CdSe) or cadmium telluride (CdTe) with a zinc sulfide (ZnS) shell. Their excellent spectroscopic properties have enabled new applications of quantum dots in the biomedical field. However, improving the solubility of quantum dots requires efficient, chemoselective bioconjugation methods. The toxicity of CdSe QDs depends on their physicochemical properties, such as size and capping reagent, among others [48,49,50]. Therefore, efforts are underway to reduce the toxicity of CdSe QDs, including the development of a shell composed of a higher-band-gap, less toxic material, such as ZnS or silica, over the emissive semiconductor nanocrystal core (CdSe or CdTe), thereby creating core/shell nanocrystals [50].

Furthermore, the breath figure method, which uses water droplet templates to create porous films, has attracted significant interest due to its simplicity and wide range of applications. However, further research is still needed on new materials, advancements in preparation technologies for controlling nano- and microstructures, and large-area fabrication [51].

The ability to scale up honeycomb film production could significantly expand their potential in fields such as optics, photonics, surface science, biotechnology, and regenerative medicine [51,52,53,54,55,56].

Connal and coworkers fabricated metal-containing honeycomb films from spiropyran polymers [57]. Connal et al. have demonstrated that a spiropyran-based functional polymer can be synthesized and used to form highly ordered honeycomb materials via the breath figure technique, which relies on the self-assembly of water droplets. These materials undergo rapid, intense color changes both in solution and as porous films upon irradiation with light (UV or visible).

Our research group previously reported on a series of hybrid polymer films and their optical, morphological, and wettability properties. This study involved adding fluorescent ZnS nanoparticles into polymer matrices to modify their optical and thermal characteristics. As a result, significant changes in the optical properties of the hybrid materials were also observed [58,59,60]. Furthermore, the properties of materials result from a complex interaction among surface structure, morphology, and their physical and chemical traits. Functional surfaces with specific wettability have attracted significant interest due to their substantial benefits across various applications [61].

This research aims to make a significant contribution to the development, characterization, and application of a photoactive switch-on/-off system for potential use in biomedicine. In this study, we designed photoactive materials based on dynamic interactions between a functional photochromic component in photoactive polymers and fluorescent QDs nanoparticles serving as fluorescent agents. In this work, we highlight the following key contributions: Spiropyran-based photoactive copolymer structures are used to monitor QD nanoparticles that can switch the resonance forms of **MC**, providing a detailed discussion on an inorganic species detectable under specific experimental conditions. Additionally, in this study, we investigate the photosensitive ability of spiropyran hydrogel films in interacting with QDs. It examines the optical and structural interactions between the two components (MC-QDs) under UV-Vis radiation and through FTIR spectroscopy, respectively, and their effects on the optical and morphological properties of switch systems for biological applications. Hybrid hydrogel porous films were fabricated via a one-step bio-inspired process using the breath figure (BF) method. Therefore, we suggest that these photoactive polymer materials can serve as an alternative switch-on/off system in optoelectronics, providing a versatile platform for detecting inorganic target molecules in devices that both emit and detect light. This field is crucial to a range of applications, including LED devices, solar cells, and fiber optics. The resulting compounds were analyzed using 1H NMR, FTIR, UV-Vis, and fluorescence spectroscopy. Furthermore, changes in the chemical structure of the photoactive polymer are expected to influence its optical and morphological properties, which were assessed.

## 2. Results and Discussion

**Figure 1:**
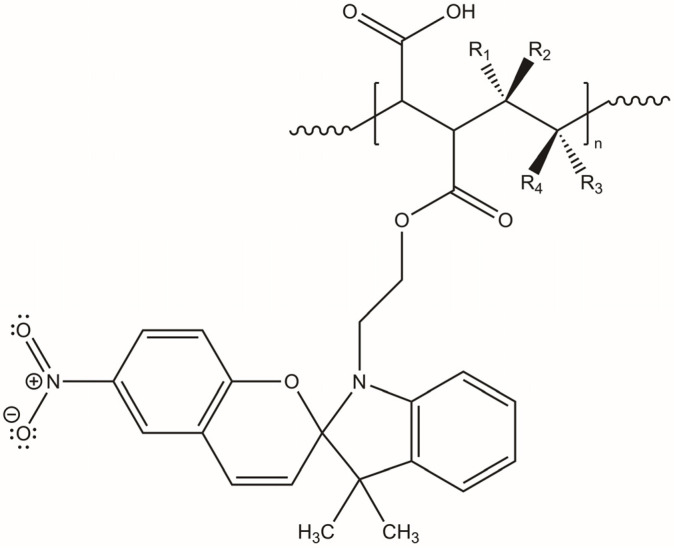
Structure of the photoactive P(MAn-alt-2MB)-**SP** (R_1_ = CH_3_; R_2_ = CH_3_; R_3_ = H; R_4_ = CH_3_) and P(MAn-alt-OD)-**SP** (R_1_ = H; R_2_ = H; R_3_ = H; R_4_ = CH_2_-(CH_2_)_16_CH_3_).

The photoactive P(MAn-alt-OD)-***SP*** and P(MAn-alt-2MB)-***SP***, see Figure 1, were obtained using the experimental procedure described elsewhere [62]. The yields of photoactive copolymers were 65.0% and 73.0%, respectively, and molecular weights of the copolymers 19,300 and 17,900, along with polydispersity indices of 1.53 and 1.78, were measured.

### 2.1. Characterization by FT-IR

**Figure 2:**
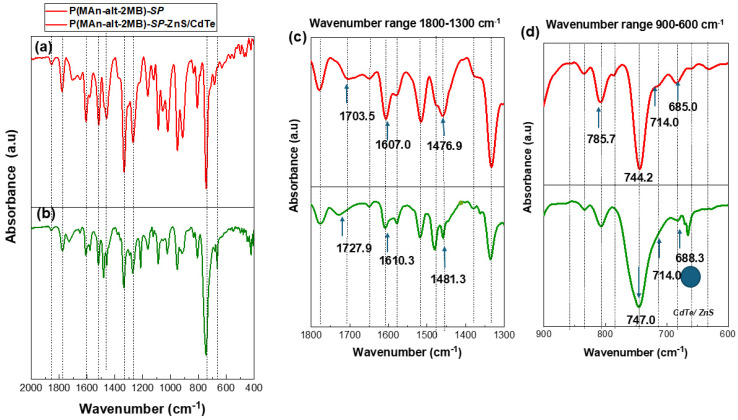
(**a**) FT-IR spectrum of P(MAn-alt-2MB)-**SP** in the wavenumber range between 2000 and 400 cm^−1^, (**b**) P(MAn-alt-2MB)-**SP** functionalized with CdTe/ZnS) **SP** in the wavenumber range between 2000 and 400 cm^−1^, (**c**) FT-IR spectrum in the wavenumber range between 1800 and 1300 cm^−1^, (**d**) FT-IR spectrum in the wavenumber range 900–600 cm^−1^.

**Figure 3:**
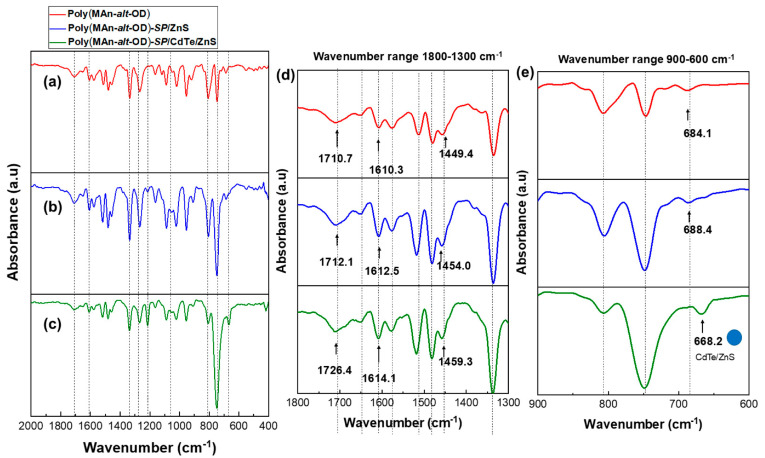
(**a**) FT-IR spectrum of P(MAn-alt-OD)-**SP**, (**b**) P(MAn-alt-OD)-**SP** functionalized with ZnS (NPs), (**c**) P(MAn-alt-OD)-**SP** functionalized with CdTe/ZnS (NPs) in the wavenumber range between 2000 and 400 cm^−1^, (**d**) FT-IR spectrum in the wavenumber range between 1800 and 1300 cm^−1^, and (**e**) FT-IR spectrum in the wavenumber range between 900 and 600 cm^−1^.

ATR-FTIR spectra were obtained using a Perkin-Elmer Spectrum-Two spectrometer equipped with a UATR accessory. The sample was placed directly on the diamond surface and scanned from 4000 to 500 cm^−1^ with a resolution of 1 cm^−1^. Table 1 summarizes the primary vibration bands in the FT-IR spectra of the photoactive copolymer films with CdTe/ZnS (QDs) functionalization, as shown in Figure 2 and Figure 3.

The most prominent signals in the FT-IR spectrum in the wavenumber range 2000–400 cm^−1^ for P(MAn-alt-2MB)-SP and P(MAn-alt-2MB)-SP functionalized with CdTe/ZnS QDs (see Figure 2a and Figure 2b, respectively) are observed at 1703.5 cm^−1^ (C=O stretching; -COOR) and at 1727.9 cm^−1^ upon functionalization with CdTe/ZnS QDs; at 1607.0 cm^−1^ (C=C stretching; Ar) and at 1610.3 cm^−1^ with CdTe/ZnS(QDs); at 1476.9 cm^−1^ (N–O stretching; -N=O) related to the SP agent and at 1481.9 cm^−1^ after interaction with CdTe/ZnS QDs.

Subsequently, these signals confirm an interaction between the **SP** moiety of the photochromic agent and the ZnS/CdTe nanoparticles, with shifts to higher wavenumber, at 1727.9 cm^−1^, 1610.3 cm^−1^, and 1481.3 cm^−1^, respectively. These signals are best observed in the FT-IR spectrum within the wavenumber range of 1800–1300 cm^−1^, see Figure 2c. Furthermore, Figure 2d shows a slight variation in wavenumber observed in the signals at 785.7 cm^−1^, 744.2 cm^−1^, 714.0 cm^−1^, and 685.0 cm^−1^, at 747.0, 714.0 cm^−1^, and 668.3, respectively, related to the interaction between the nitro groups and the CdTe/ZnS(QDs) in the wavenumber range between 900 and 600 cm^−1^.

The FT-IR spectra in the wavenumber range of 2000–400 cm^−1^ for P(MAn-alt-OD)-SP and P(MAn-alt-OD)-SP functionalized with ZnS (NPs) and CdTe/ZnS (QDs) show similar signals to the previously analyzed system, see Figure 3a, Figure 3b, and Figure 3c, respectively. These signals appear at 1710.7 cm^−1^ (C=O stretching; -COOR), 1610.3 cm^−1^ (C=C stretching; Ar), and 1449.4.9 cm^−1^ (N-O stretching; -N=O), related to the SP agent and its interaction with ZnS (NPs) and CdTe/ZnS(QDs). In these interactions, the signals shift to 1726,4 cm^−1^, 1614.1 cm^−1^ (C=C stretching; Ar), and 1459.3 cm^−1^ (N-O stretching; -N=O), with the latter also observed in the CdTe/ZnS(QDs) sample. These signals are most clearly visible in the FT-IR spectrum within the wavenumber range of 1800–1300 cm^−1^, see Figure 3d. Additionally, Figure 3e shows slight variations in the wavenumber at 684.1 cm^−1^, 688.4 cm^−1^, and 668.2 cm^−1^, which relate to the interaction between nitro groups and the CdTe/ZnS(QDs) in the 900–600 cm^−1^ range.

### 2.2. X-Ray Diffraction (XRD) of Photoactive Copolymers with CdTe/ZnS (QDs)

**Figure 4:**
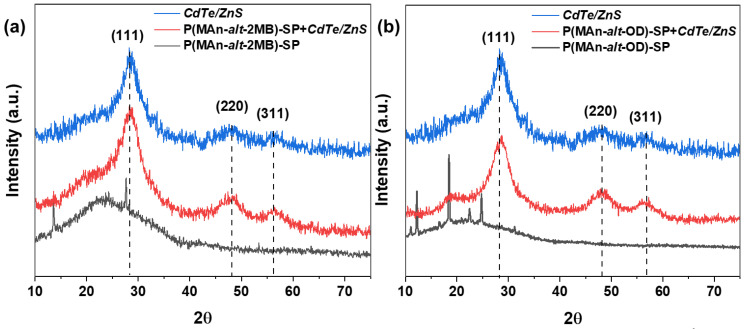
(**a**) XRD patterns of P(MAn-alt-2MB)-***SP***; P(MAn-alt-2MB)-***SP***+QDs and CdSe/ZnS (QDs), (**b**) XRD patterns of P(MAn-alt-OD)-***SP***; P(MAn-alt-OD)-***SP***+QDs and CdSe/ZnS (QDs).

Figure 4 shows the XRD patterns of (a) P(MAn-alt-2MB)-SP; P(MAn-alt-2MB)-SP+CdTe/ZnS(QDs), CdTe/ZnS(QDs), and (b) XRD patterns of P(MAn-alt-OD)-SP+CdTe/ZnS(QDs) and CdTe/ZnS(QDs) with a series of diffraction peaks in the 2θ range (10–70°), indicating the presence of the QDs and their interaction with the photoactive copolymers. XRD patterns Figure 4a show evidence that the CdTe/ZnS QDs exhibit three strong diffraction peaks at 2θ = 28.46°, 47.64°, and 56.31°, corresponding to the characteristic (111), (220), and (311) planes of the standard zinc blende structure of ZnS. This confirms that ZnS layers have been successfully deposited on the CdTe core. Additionally, QDs are observed in the diffraction patterns of both photoactive polymer matrices. Figure 4b XRD patterns show the characteristic zinc blende planes (111), (220), and (311) appearing at 28.16°, 48.51°, and 56.63° for CdTe/ZnS, within the 10–70° 2θ range. The positions of the XRD peaks of the CdTe cores closely match those of bulk CdTe with a cubic structure [59]. After the growth of the ZnS shell on the CdTe core, the peak positions shift to higher angles toward the peaks of bulk ZnS cubic structures, which confirms the formation of CdTe/ZnS core–shell particles [59,63]. In both diffractograms, the polymer matrices exhibit a diffuse diffractogram, due to an optical phenomenon characterized by a circle, or ring of faint light in the diffractogram instead of sharp peaks, which indicates short-range order or molecular disorder; that is, they are amorphous in nature

### 2.3. Characterization by UV-Vis, Optical Fluorescence Microscope, and SEM-DSX Microscopy

**Figure 5:**
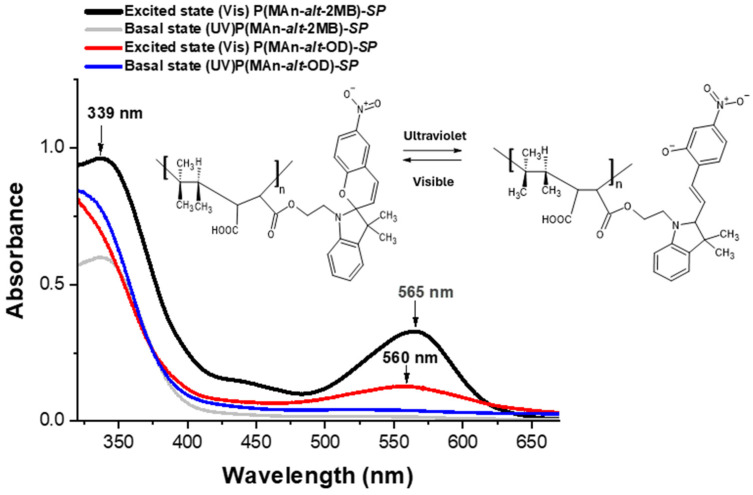
UV-Vis spectra of photoactive-copolymer solution (1 g·L^−1^, in CHCl_3_), before and after irradiation at 365 nm.

The photoactive copolymer-**SP** solutions showed photoinduced reversible interconversion upon UV-Vis irradiation (365 nm) in THF (see Figure 5). The photoinduced formation of the colored state, “merocyanine” (**MC**), is responsible for the significant increase in absorbance at approximately 560 nm in the visible region.

Figure 5 illustrates the effect of irradiating the copolymers at 365 nm on the absorption spectrum. Before UV irradiation (in the basal state), the copolymers exhibit an absorption peak at 340 and 339 nm. After irradiation, the photoactive copolymers exhibit a maximum at 565 and 560 nm (in the excited state), indicating the photoconversion from **SP** to **MC** (in an open conformation) [4,64]. In principle, ultraviolet irradiation induces the corresponding isomerization **MC**, characterized by an absorption band at 565 nm for (MAn-alt-2MB).

### 2.4. Interaction of the Components -SP-QDs Under UV-Vis Radiation

**Figure 6:**
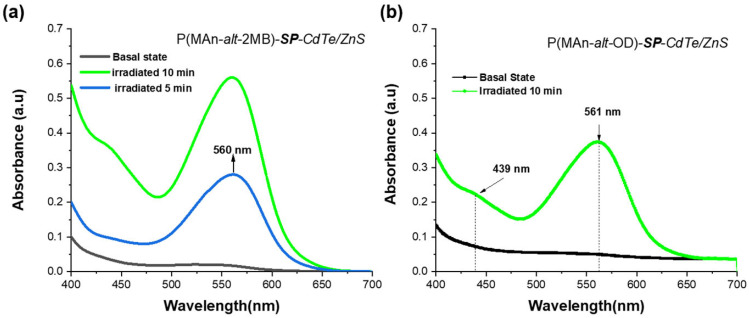
(**a**) Absorption spectra of photoactive polymer-CdTe/ZnS (NPs) in solution (1 g·L^−1^, in CHCl_3_) after UV irradiation, (at 365 nm, 0.5 mW/cm^2^) for P(MAn-alt-2MB)-***SP*** for 5 and 10 min; (**b**) for P(MAn-alt-OD)-***SP*** for 10 min.

**Figure 7:**
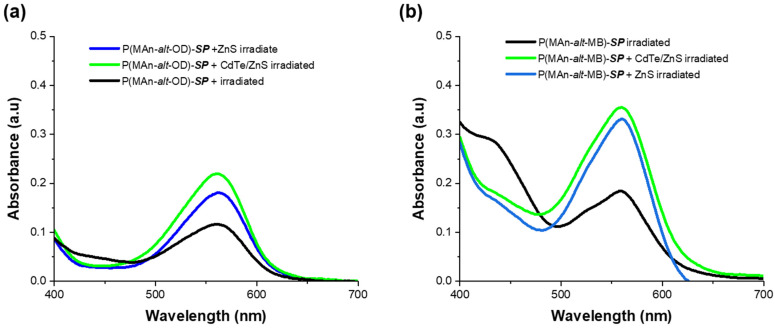
(**a**) Absorption spectra of P(MAn-alt-OD)-SP with ZnS (NPs) and CdTe/ZnS (QDs) with ZnS (NPs) and with CdTe/ZnS (QDs) in solution (1 g·L^−1^, in CHCl_3_), after 15 min of irradiation; (**b**) P(MAn-alt-2MB)-SP with ZnS (NPs) and CdTe/ZnS (QDs) with ZnS (NPs) after 15 min of UV irradiation (at 365 nm, 0.5 mW/cm^2^).

The Tauc equation is used in the ultraviolet (UV), visible (Vis), and sometimes near-infrared (NIR) regions. This is because photons in these ranges have enough energy to excite an electron from the valence band to the conduction band. The samples were illuminated using a lamp that emitted light from 200 nm (UV) to 800–1100 nm (Visible/NIR). Figure 6 and Figure 7 show the absorbance and wavelength in the 400–700 nm range, corresponding to the visible region of the spectrum. Figure 6a,b show the absorbance after irradiation for 5 min and 10 min in the presence of ZnS (NPS) and CdTe/ZnS (QDs). The P(MAn-alt-2MB)-SP with ZnS (NPs) and CdTe/ZnS (QDs) shows that the absorbance of the ***SP*** moiety at 560 nm increases; however, for the P(MAn-alt-OD)-SP with ZnS (NPs) and CdTe/ZnS (QDs) system, no changes in the absorption were observed after 5 min of irradiation.

Figure 7a shows the absorbance for the P(MAn-alt-OD)-***SP*** with ZnS (NPs) and CdTe/ZnS (QDs) after irradiation for 10, 15, and 25 min. Figure 7b The P(MAn-alt-2MB)-SP with ZnS (NPs) and CdTe/ZnS (QDs) after irradiation for 15 min, shows that the absorbance of the ***SP*** moiety at approximately 560 nm, increases; however, for the P(MAn-alt-OD)-***SP*** with ZnS (NPs) and CdTe/ZnS (QDs), the increase was smaller.

The absorption spectrum was attributed to the interaction between the ZnS/CdTe nanoparticles and the photoactive polymer, leading to energy transfer from the polymer to the nanoparticles [4].

The band gaps of the systems, including photoactive copolymers and ZnS/CdTe QDs, were determined (see Table 2). The optical band gap Eg was estimated from UV-Vis measurements for irradiation at 254 and 365 nm wavelengths, using the Tauc equation [64]:

(1) $$(\alpha h\nu )={A(h\nu -Eg)}^{m}$$

where α is the absorption coefficient, h is Planck’s constant, ν is the frequency of light, A is a constant, m is a constant related to the type of optical transition, and Eg is the band gap. The absorption coefficient is estimated from α = 2.303 A/d, A is the absorbance, and d is the compactness of the film [65]. The value of m = 1/2 was used in the calculations because it was considered a direct optical transition for all the compounds; therefore, (αhν)^2^ versus hν, the photon energy, was plotted. The linear part of the figure was extrapolated to (αhν)^2^ = 0 using a straight line to estimate the band gap. The estimated Eg values are shown in Table 2, indicating that after irradiation at λ = 365 nm, the estimated band gap for photoactive copolymers-**SP** is slightly lower than that of ZnS, at 3.95 eV. The ZnS band gap is close to the reported maximum and exhibits a blue shift relative to the literature [65]. This is due to quantum confinement in nanocrystalline ZnS thin films. After irradiation at λ = 365 nm for 5, 10, 15, 20 and 25 min, the band gap for P(MAn-alt-OD)-**SP**-(NPs) and P(MAn-alt-2MB)-**SP**-(NPs) decreased. This decrease in Eg value is attributed to the interaction between the NPs and the **SP** moiety surface, resulting from direct binding in the open-ring state, which is in contact with the inorganic nanoparticles.

**Figure 8:**
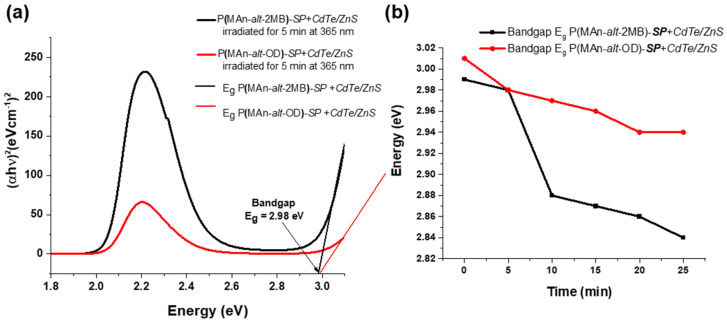
Shows the band gaps for the P(MAn-alt-2MB)-**SP**-CdTe/ZnS(NPs) and P(MAn-alt-OD)-**SP**-CdTe/ZnS(NPs) when exposed to visible light at 365 nm, (**a**) for 5 min and (**b**) for 5, 10, 15, 20 and 25 min.

For the composites, photoactive copolymers –**SP**-QDs, the band gap is lower than for the NPs (see Table 2 and Figure 8). When exposed to visible light at 365 nm for 5 min, the band gaps of P(MAn-alt-OD)-**SP**-CdTe/ZnS (NPs) and P(MAn-alt-2MB)-**SP**-CdTe/ZnS(NPs) are slightly reduced to 3.07 and 2.89 eV, respectively, which are lower in the presence of the NPs, as shown in Table 2. The reduction in the band gap could be due to the formation of a new transitional level below the conduction band [66]. Therefore, the **SP** moiety combined with the NP QDs could promote the formation of this new transition level.

### 2.5. Fluorescence Studies by Spectroscopy

**Figure 9:**
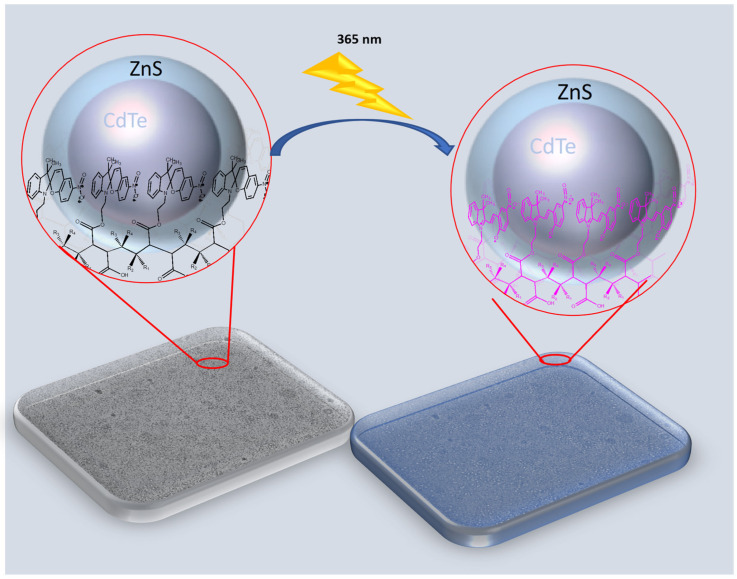
Schematic illustration of a fluorophore–photochrome dyad interacting under ultraviolet light, which can cause the photochrome to switch states and activate an energy transfer pathway between components.

The photoinduced interconversion between the two states of a photochromic compound **(SP ↔ MC)** can be used to control the emission of fluorescent QDs (CdTe/ZnS). In fact, both fluorescent and photochromic components can be combined into a single molecular structure, and the photochemical changes to the latter can be engineered to regulate the emission of the former. For example, ultraviolet irradiation of a fluorophore–photochrome dyad (Figure 9) can cause the photochrome to switch between states, thereby activating an electron- or energy-transfer pathway between components.

These pathways can quench the fluorophore’s excited state, effectively turning off its emission. Conversely, thermal or photochemical regeneration of the original photochrome form can stop the quenching and restore the fluorophore’s emission. Therefore, the emissions of these fluorophore–photochrome dyads can be repeatedly turned on and off by modulating their photochromic component between its two states with optical stimulation.

**Figure 10:**
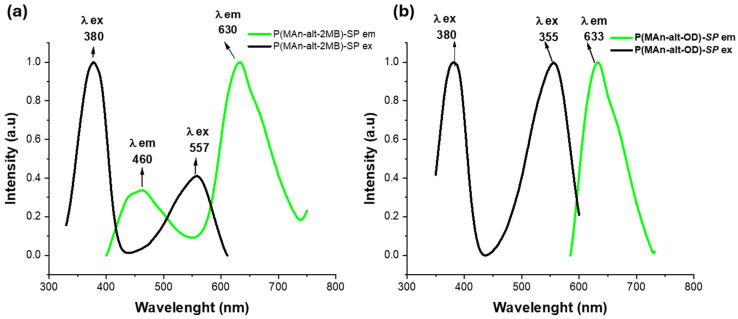
Shows the excitation and emission bands of (**a**) the photoactive P(MAn-alt-OD)-**SP**; (**b**) the photoactive P(MAn-alt-2MB)-**SP** (1.0 × 10^−3^ mg mL^−1^) after 10 min UV irradiation (λ exc. = 390 nm).

After UV light irradiation, as shown in Figure 10, both photoactive polymers turn purple due to the merocyanine (**MC**) isoform. It is possible to observe their optical spectra for both photoactive copolymers. For P(MAn-alt-OD)-***SP***, an emission band at 633 nm can be observed, as shown in Figure 10a. Conversely, for P(MAn-alt-OD)-**SP**, with an excitation band at 380 nm and 556 nm, there is a single emission band at 633 nm, indicating a single **MC** conformation. On the other hand, for P(MAn-alt-2MB)-**SP**, two emission bands were observed at 470 and 610 nm (λ = 390 nm), along with an absorption band at 380 nm, as seen in Figure 10b. These two emission signals correspond to different conformations of the **MC**, with two interconvertible structures in equilibrium, in which reverse isomerization occurs spontaneously and can be accelerated by exposure to visible light. The double-bond-containing **MC** isomer has several resonance forms, each with different optical spectra [66].

**Figure 11:**
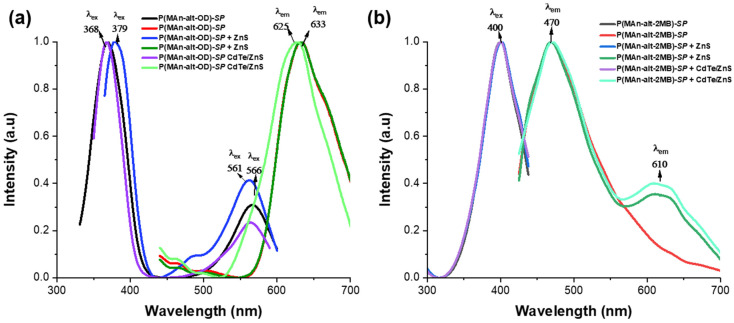
Shows the excitation and emission bands of the photoactive polymers (**a**) P(MAn-alt-OD)-**SP** +QDS and (**b**) P(MAn-alt-2MB)-**SP** + QDs, in chloroform solution, after irradiation.

Figure 11a displays the excitation and emission bands of both photoactive P(MAn-alt-OD)-**SP** +QDS in chloroform solution after irradiation at λexc = 390 nm. The influence of QDs (NPs) on the photoluminescent properties of the copolymers can be explained by energy transfer. Specifically, the excitation energy of the QDs (NPs) can be transferred to the photogenerated state **MC** when the components are sufficiently close. This emission signal corresponds to one resonance form conformation of the **MC**, with two interconvertible structures in equilibrium, each displaying a distinct optical spectrum. Figure 11a shows a single emission band at 625 nm for both P(MAn-alt-OD)-**SP** + ZnS (NPs) and at 633 nm for P(MAn-alt-OD)-**SP** + CdTe/ZnS (QDs). Consequently, the excitation energy of the CdTe/ZnS (NPs) can be transferred to the photogenerated state **MC** of the photoactive polymer when both components are close enough. Meanwhile, Figure 11b demonstrates the effect of CdTe/ZnS NPs on the photoluminescent properties of the photoactive P(MAn-alt-2MB)-**SP**. After excitation at λexc = 390 nm, the polymer P(MAn-alt-2MB)-**SP** exhibits two emission bands at 470 nm and 610 nm, with the emission band of excited CdTe/ZnS nanoparticles (NPs) overlapping with the photogenerated state **MC** at 610 nm. Consequently, the excitation energy of the CdTe/ZnS (NPs) cannot be transferred to the photogenerated state **MC** of the photoactive polymer at 470 nm because the two components are not close enough. These two emission signals correspond to different conformations of the **MC**, with two interconvertible structures in equilibrium, where reverse isomerization occurs spontaneously and can be accelerated by exposure to visible light. The double-bond-containing **MC** isomer has several resonance forms, each with a different optical spectrum. Similarly, the emission spectrum of P(MAn-alt-2MB)-**SP** + CdTe/ZnS displays prominent luminescence bands at 470 nm and a lower-intensity band at 610 nm, originating from different resonance-form conformations of the **MC**, which are in equilibrium with two interconvertible structures with distinct optical spectra. Additionally, the reversible changes in the absorbance and fluorescence spectra of **MC** are affected by the environment [4,66]. The analysis of the interaction between CdTe/ZnS nanoparticles (Quantum Dots—QDs) and spiropyran-derived photochromic agents reveals key limitations that affect the efficiency of luminescence control. These limitations mainly involve kinetic, energetic, and structural factors. First, Resonance Energy Transfer (FRET) requires sufficient spectral overlap between the QD emission (donor) and the absorption of the colored spiropyran form (merocyanin, acceptor). If the QD emission peak does not match the merocyanin absorption maximum (usually 540–580 nm), the transfer efficiency drops significantly. Additionally, Photoinduced Electron Transfer (PET) occurs when spiropyran changes to merocyanine, altering its reduction potential and activating a competing electron-transfer pathway that can quench fluorescence, even when FRET is inefficient. The coexistence of these processes complicates the model predictions. Steric limitations related to the QD surface also contribute. The structure of the CdTe/ZnS QD can hinder the transformation of spiropyran to merocyanine, especially if the photochromic agent is densely packed near the ZnS shell. Moreover, stabilizing ligands can increase the interaction radius, preventing effective energy or electron transfer [35,36,63]. In summary, the success of the design depends on precisely tuning redox potentials and spectral overlaps, as well as creating an environment that allows spiropyran to undergo conformational change without interference from the QD surface. In the context of the interaction between CdTe/ZnS quantum dots (QDs) and spirane derivatives, the concept of the band gap is fundamental. However, its influence does not arise from a physical change in the semiconductor’s band gap, but rather from the position of the photochrome’s energy states relative to the QD’s bands. The band gap must be tuned (via particle size) so that the exciton (electron-hole) relaxation energy matches the energy needed to excite the merocyanine form of the spirane. If the band gap is too wide (causing blue/UV emission) or too narrow (causing IR emission), the overlap with the merocyanine orbitals is lost, making the FRET mechanism inefficient. The most critical interaction with the band gap occurs through photoinduced electron transfer (PeT). When the spirane converts to merocyanine, its molecular levels (HOMO and LUMO) shift, and intermediate levels appear. Merocyanine creates an intermediate energy level within or near the QD’s band gap. Upon QD excitation, instead of the electron in the conduction band (CB) returning to the valence band (VB) to emit light, the electron jumps to the LUMO level of merocyanine because it is lower in energy and more favorable. Consequently, the “effective” band gap of the entire system is short-circuited by the photochrome, which prevents luminescence.

In summary, the intrinsic band gap of CdTe/ZnS (QDs) remains unchanged; however, the presence of merocyanine introduces energetic “shortcuts” (competing energy levels) that intercept electrons before they can fluoresce, ultimately dominating the material’s optical response [4,64].

### 2.6. Characterization by Fluorescence Optical Microscope and SEM

**Figure 12:**
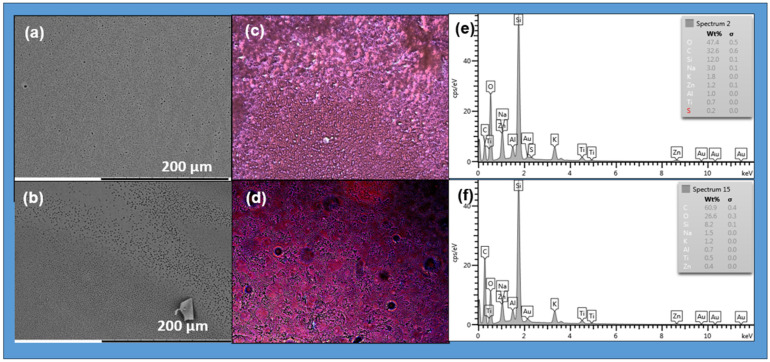
SEM images for the photoactive (**a**) P(MAn-alt-2MB)-**SP**+ZnS and (**b**) P(MAn-alt-OD)-**SP**+ZnS at 3 g·L^−1^ and 2 wt% of QDs (NPs) in CHCl_3_, respectively: (**c**,**d**) Optical microscope images of the photoactive copolymers after irradiation respectively; (**e**,**f**) SEM-EDX (energy dispersive X-ray spectroscopy) reveals the elemental makeup of the surfaces and the chemical composition for the photoactive P(MAn-alt-2MB)-**SP** and P(MAn-alt-OD)-**SP** at 2 wt% of ZnS, respectively.

The photoactive copolymers can be functionalized with ZnS or CdTe/ZnS nanoparticles to create hybrid materials with improved properties for various applications, including optoelectronics, biomedicine, and electronics. Determining the photoluminescence properties of the photoactive polymer is crucial for understanding its role in the self-assembled system. The SEM micrograph of the prepared films, see Figure 12a,b, shows both photoactive copolymers with NPs, P(MAn-alt-2MB)-**SP** +ZnS (NPs) and P(MAn-alt-OD)-**SP** + ZnS (NPs), in chloroform solution. The SEM images display self-assembled layers with a porous surface, resulting from the systems’ tendency to reach a hydrophilic-hydrophobic balance during self-assembly. To compare, the optical images of the surface of photoactive copolymers P(MAn-alt-2MB)-**SP**+ ZnS (NPs) and P(MAn-alt-OD)-**SP**+ZnS (NPs), at 1 g·L^−1^, in the presence of ZnS (NPs) (2 wt%), were taken under natural light and after UV irradiation (see Figure 12c,d). Incorporating ZnS (NPs) into the photoactive P(MAn-alt-2MB)-**SP** and P(MAn-alt-OD)-**SP** promotes violet color emission after UV exposure. Meanwhile, SEM images suggest the presence of ZnS nanoparticles within the **SP** photoactive copolymer, which induce its self-assembly, promote interaction between the two components, and lead to the formation of a porous surface. The transformation of the -**SP** isoform into the -**MC** form within the polymer matrix, which then interacts with the nanoparticles, results in a switch in the blue luminescence. Because of their optical properties, these materials are promising for the fabrication of light-sensitive porous films. Using the breath figure (BF) technique, hexagonal films form under favorable lighting conditions in the presence of NPs.

The film micrographs indicate that the inorganic component is well dispersed within the photoactive copolymer systems, with a uniform, homogeneous distribution of ZnS NPs across the phases. Furthermore, the colors observed in the optical microscope images, both before and after irradiation, are due to the light emitted by the components in the composite films. The inset EDX spectrum in Figure 12e,f shows the elemental composition of the corresponding image. In addition to the standard peaks of carbon and oxygen at 0.2–0.5 keV from the polymer, other peaks at 8.5 keV (zinc) and 2.6 keV (sulfur) in the nanoparticles are observed.

**Figure 13:**
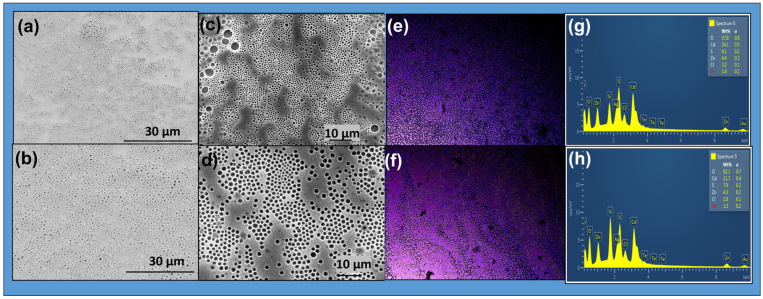
SEM and optical microscope images for the photoactive P(MAn-alt-2MB)-**SP**+CdTe/ZnS and P(MAn-alt-OD)-**SP**+CdTe/ZnS at 1 g·L^−1^ and 2 wt% of QDs (NPs) respectively (**a**–**d**) in CHCl_3_. Fluorescence after irradiation at 365 nm (**e**,**f**) for the photoactive P(MAn-alt-2MB)-**SP** and P(MAn-alt-OD)-**SP** at 2 wt% of CdTe/ZnS, respectively. SEM-EDX images (**g**,**h**) for the photoactive P(MAn-alt-2MB)-**SP** and P(MAn-alt-OD)-**SP** at 2 wt% of QDs (NPs), respectively.

Figure 13 shows SEM images of the films prepared with CdTe/ZnS (NPs). It also displays optical microscope images of photoactive copolymers with CdTe/ZnS NPs at 2% wt. after UV irradiation at λ = 365 nm. All the films exhibit violet and blue colors under fluorescent light. The images reveal a change in the photoactive films from the isoform **SP** to **MC**, and interaction with QDs (NPs) is the main factor contributing to the blue color emission. The blue luminescence was attributed to the transition from -**SP** to -**MC** and to the incorporation of QDs (NPs) into the photoactive polymer matrix, as shown in Figure 13e,f. In the presence of CdTe/ZnS (NPs), the spherical-like structures of the copolymers remain intact (Figure 13a,b). This may indicate an interaction between the photoactive copolymer and CdTe/ZnS (NPs), which prevents the self-assembly of the polymer.

It is expected that the interaction between the CdTe/ZnS (NPs) and the **SP** moiety alters the arrangement described above. Thermodynamic factors generally influence the shape of the porous surface in the film. The increase in interfacial tension, which restricts polymer chain mobility due to the coordinated CdTe/ZnS (NPs), results in a more disordered polymer film. The morphology and composition of the photoactive copolymers and their corresponding composites with CdTe/ZnS (NPs) at 2 wt% were analyzed using SEM and EDX in Figure 13g,h. The EDX spectrum shows the elemental composition of particles, including sulfur, zinc, cadmium, and tellurium. Additionally, peaks at 0.2–0.6 keV from the polymer’s carbon and oxygen, peaks at 3.0–3.8 keV for cadmium, peaks at 3.8–4.8 keV for tellurium, a peak at 8.6 keV for zinc, and a peak at 2.4 keV for sulfur in the nanoparticles are observed.

In the presence of CdTe/ZnS nanoparticles, the **MC** isoform can bind to the surface of the inorganic NPs through functional groups containing nitrogen, iminium, and oxygen from the nitro group. Emissions from the **MC** isoform can overlap with those of the QDs. Therefore, this physical interaction can facilitate energy transfer from the excited inorganic NPs to the photochrome in its photogenerated state. It can then be transferred to the photochromic component’s photogenerated state, returning it to the closed-ring isomer (spiropyran, **SP**). The conversion of the **MC** moiety into **SP** should disrupt the interaction between the functional group in the polymer chain and CdTe/ZnS NPs.

### 2.7. Measurement of Contact Angle

The wettability of the porous film surface is a crucial property that affects many practical applications, depending on the macromolecular geometric structure and the material’s chemical composition [61,67,68]. Measuring the water contact angle (WCA) provides direct insight into the sample’s wettability. The roughness and porosity of the film surface influence the contact angle values. Additionally, higher roughness increases the contact area, thereby strengthening liquid–solid interactions. For contact angles less than 90°, the surface is generally considered hydrophilic. Contact angles between 90° and 150° indicate a hydrophobic surface. When the WCA exceeds 150°, the surface is typically called superhydrophobic.

**Figure 14:**
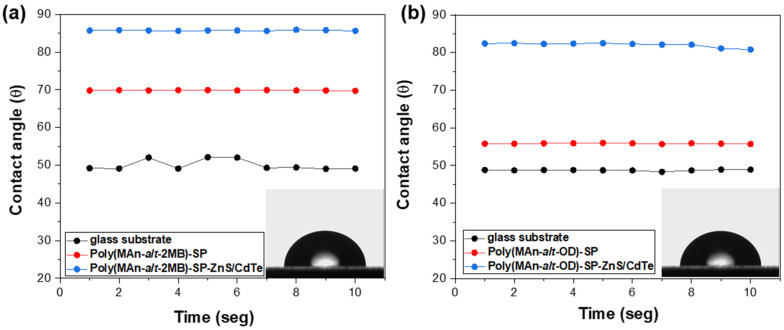
Graph of WCA of the hydrophilic films with CdTe/ZnS nanoparticles (2 wt%) on glass substrate of (**a**) P(MAn-alt-2MB)-**SP** and (**b**) P(MAn-alt-OD)-**SP**.

The WCA values obtained for the photoactive polymer films P(MAn-alt-2MB)-**SP**, P(MAn-alt-OD)-**SP**, and the CdTe/ZnS -functionalized ones ranged from 69.8° to 85.7° and from 58.8° to 82.1°, respectively. Based on WCA values, hybrid photoactive films are classified as hydrophobic (see Figure 14 and Table 3). These results can be attributed to the incorporation of inorganic functionalization, which altered the self-assembly of the macromolecules and likely affected their surface roughness. These changes could specifically enhance the hydrophobicity differences at the film surface by increasing strain due to interactions between the **SP** moiety and NPs anchored in the polymer film, on the glass substrate, and within the porous surface.

## 3. Conclusions

In this study, the photoactive components P(MAn-alt-2MB)-**SP** and P(MAn-alt-OD)-**SP** were functionalized with CdTe/ZnS QD nanoparticles and then used to create honeycomb hydrogel films with photosensitive properties after exposure to UV radiation, which, under appropriate experimental conditions, generated porous structures with photochromic stimulus–response properties. This occurs due to the inclusion of QDs nanoparticles and the presence of hydrophilic and hydrophobic domains in the photoactive copolymers. These films are sensitive to UV light, and their optical and morphological properties change after exposure. Incorporating ZnS nanoparticles or CdTe/ZnS QDs into the photoactive polymer results in violet and blue emission, respectively, upon UV exposure. The transformation of the -**SP** isoform results in the blue luminescence switching to the -**MC** form within the polymer matrix, which then interacts with the nanoparticles. Using the breath figure (BF) technique, hexagonal films are formed by creating favorable lighting conditions alongside the NPs. The results indicate that these materials could significantly contribute to the field of materials science essential for producing light-sensitive porous films. We conclude that altering the structure of the photoactive copolymers and adding QD NPs influences the surface film properties through their interactions with the matrix and the QDs. SEM analysis shows a notably porous surface in the films, which still maintains hydrophobic characteristics. This work introduces a comprehensive method for creating hybrid organic/inorganic porous films, along with a subsequent approach for fabricating quantum dot (QD)-based micropore structures using honeycomb film as a template. It is anticipated that the protocols outlined here could be further refined to enhance advanced sensor systems or develop innovative catalytic supports. This field is crucial for various applications, including LED devices, solar cells, and fiber optics.

## 4. Materials and Methods

### 4.1. Reagents

Chemicals were used without further purification and were of analytical grade. Maleic anhydride (MAn), 2-methyl-2-butene (2MB), 1-octadecene (OD), tetrahydrofuran (THF), benzoyl peroxide (BPO, 99.98%) from Sigma-Aldrich (St. Louis, MO, USA), diethyl ether, magnesium sulfate, sodium carbonate, hydrochloric acid, chloroform, acetone, dimethyl sulfoxide, and methanol were used (Merck, Stuttgart, Darmstadt, Germany). 2,3,3-Trimethylindoline, 2-bromoethanol, 2-butanone, trimethylamine, and 2-hydroxy-5-nitrobenzaldehyde were purchased from Sigma-Aldrich (St. Louis, MO, USA) and used without further purification for copolymer synthesis. 2-Hydroxy-4′-(2-hydroxyethoxy)-2-methylpropiofenone. (Irgacure 2959, 99.5%), and The *N*′*N*-methylene bisacrylamide (MBA, 99.5%) from Merck, Stuttgart, Darmstadt, Germany.

All reagents were of analytical grade from Sigma-Aldrich, including cadmium chloride (99%), mercaptopropionic acid (97%), tellurium powder (99%), and sodium borohydride (98%). Sodium sulfide [Na_2_S] flakes, zinc acetate [(CH_3_COO)_2_Zn·2H_2_O], and potassium dihydrogen orthophosphate [KH_2_PO_4_] were purchased from Merck (Stuttgart, Germany). Cadmium acetate dihydrate [(CH_3_COO)_2_Cd·2H_2_O] was obtained from Sigma-Aldrich (Sigma-Aldrich Química Ltd., Santiago, Chile), along with potassium hydroxide (KOH). Sodium hydroxide [NaOH] and sodium sulfite anhydrous [Na_2_SO_3_] were sourced from Merck (Stuttgart, Germany). No further purification was performed before synthesizing the copolymers.

### 4.2. Measurements

The structures of the polymer and photoactive polymers were determined by proton nuclear magnetic resonance (^1^H-NMR) on a Bruker 400 MHz spectrometer (Karlsruhe, Germany). Gel permeation chromatography (GPC) was performed to measure the number-average molecular weight (Mn) and weight-average molecular weight (Mw) under the following conditions: a Waters 600E instrument (Milford, MA, USA) equipped with UV and RI detectors, using DMF as the solvent. ATR-FTIR spectra were obtained using a Perkin-Elmer Spectrum-Two spectrometer (Waltham, MA, USA) equipped with a UATR accessory. The sample was placed directly on the diamond surface and scanned from 4000 to 500 cm^−1^ with a resolution of 1 cm^−1^. X-ray Diffraction (XRD) analysis was performed using a Bruker D-2 Phaser equipped with CoKα radiation (λ = 1.7902 Å), with a step size of 0.05° and a step time of 5 s, over an angular range of 10 to 90° 2θ, (Karlsruhe, Germany). The morphological properties of the photoactive block copolymer and the photoactive polymer functionalized with ZnS and CdTe/ZnS QDs surface hydrogel films were analyzed using optical fluorescence microscopy and scanning electron microscopy (SEM) with energy dispersive X-ray spectroscopy (SEM-EDX), utilizing a Zeiss EVO MA 10 scanning electron microscope equipped with an EDX Penta FET precision detector from Oxford Instruments, (Thermo Fisher Scientific), Originally based in Eindhoven, The Netherlands. The EDX detector system is often produced by EDAX, headquartered in Mahwah, NJ, USA, located at the Center for the Development of Nanoscience and Nanotechnology, Chile. UV-Vis spectra were recorded on a Perkin-Elmer Lambda-35 spectrophotometer (Perkin-Elmer, Waltham, MA, USA) with a resolution of 1 nm, using quartz cuvettes with a 1 cm path length. Fluorescent spectra were recorded on a Shimadzu RF-5301PC spectrofluorophotometer (Shimadzu, Kioto, Japan) with a resolution of 1 nm.

A LEICA Model DM 2000 LED optical microscope was used with a LEICA MFC 170 HD camera, (Wetzlar, Germany). The camera was set to an automatic exposure of 500.00 ms and a saturation of 120. The image surface measured 549.45 μm × 412.09 μm. For fluorescence imaging, the aperture was set to 1/3 and the focus to 3/3. The hydrogel films were prepared using a Darwin model PH 9- DA chamber with a temperature of 25 °C. Spin-coating (Chemat Scientific, coupled with an oil-free vacuum pump, Rocker Chemker 410), by Rocker Scientific Co., Ltd., (Kaohsiung, Taiwan).was used to deposit the copolymer solutions onto glass substrates Fourier transform infrared spectroscopy (FTIR) spectra were acquired by a Bruker Vector 22 (Bruker Optics GmbH, Karlsruhe, Germany).

Contact angle measurements were performed using an optical tensiometer (Ramé-Hart model 250-F4, Succasunna, NJ, USA) with a temperature controller and automatic volume dispenser, and a sessile drop-on-drop method over a solid substrate—software DROP image Advanced (Ramé-Hart Inc., Succasunna, NJ, USA).

### 4.3. Synthesis and Characterization of ZnS and CdTe (QDs)

#### 4.3.1. Synthesis and Characterization of ZnS (QDs)

Our group previously reported the procedure for synthesizing ZnS nanoparticles and their characterization using an X-ray diffractometer [59]. ZnS (NPs) are synthesized through a simple chemical co-precipitation method at room temperature, with the pH value varied. The XRD pattern results indicate the presence of a wurtzite ZnS crystalline phase (hexagonal) [59,69]. The diffraction peaks between 30 and 70° match the wurtzite ZnS crystalline phase. For example, sharp, intense peaks assigned to the (100), (002), and (101) reflections from wurtzite ZnS occur between 20° and 30°, suggesting that the sample is finite. The average crystallite size was estimated to be 8–10 nm using Scherrer’s equation.

#### 4.3.2. Preparation of CdTe QDs

Aqueous colloidal CdTe QDs were synthesized using the following methodology: In a three-neck round-bottom flask fitted with a condenser, cadmium chloride (215 mg, 1.17 mmol) was dissolved in 60 mL of water with stirring. Mercaptopropionic acid (133 µL, 1.52 mmol) was added, and the pH was adjusted to 11.0 with 0.1 mol/L NaOH. The solution was thoroughly deoxygenated with argon for 30 min, after which freshly prepared NaHTe (0.58 mmol, 880 µL) was introduced under stirring and a gentle argon flow. The reaction mixture was heated to reflux, and the formation of the QDs was monitored by recording UV-Vis and fluorescent spectra of samples taken at specific reaction times. These samples were dialyzed using a Spectrapore^®^ dialysis membrane (cutoff 14 kDa), (Rancho Dominguez, CA, USA) to remove excess polymer and reaction chemicals. Telluride precursor (NaHTe) was prepared by reducing tellurium with sodium borohydride under an argon atmosphere, as reported elsewhere [70,71]. The diameter of QDs was measured using TEM. The QDs prepared at 60 min had a diameter of 3.2 ± 0.5 nm. This matches the results from the empirical equation proposed by Peng et al. [72].

#### 4.3.3. Procedures for Obtaining CdTe/ZnS (QDs)

Thioglycolic acid (TGA) encapsulated ZnS/CdTe QDs were synthesized using the method described [73,74,75]. CdTe/ZnS core/shell NPs were prepared by the controlled growth of ZnS over the CdTe quantum dots (QDs) surface. For this, 10 mL of CdTe QDs were placed in a round-bottom flask, and 0.05 M Na2S and 0.05 M (CH_3_COO)_2_Zn·2H_2_O solutions were added alternately in small aliquots with continuous stirring at room temperature. Furthermore, acetone was added to separate the NPs from unreacted chemicals and by-products. The NP precipitates were filtered, then washed several times, and finally redispersed in water at different concentrations to study the interactions.

#### 4.3.4. Procedure of Synthesis for Photochromic Agent

The synthesis of the photochromic agent 1-(2-hydroxyethyl)-3,3-dimethylindoline-6-nitrobenzopyran (**SP**) occurs in two steps. The first step involves synthesizing 1-(2-hydroxyethyl)-2,3,3-trimethylindolenine bromide salt. In the second step, 1-(2-hydroxyethyl)-3,3-dimethylindoline-6-nitrobenzopyran was carried out in a 100 mL three-neck flask equipped with a condenser and a magnetic stirrer, by combining 1 g (3.52 mmol) of 1-(2-hydroxyethyl)-2,3,3-trimethylindolenine bromide, 0.6 g (3.52 mmol) of 2-hydroxy-5-nitrobenzaldehyde, 2 mL of triethylamine (14.08 mmol), and 10 mL of ethanol. The mixture was heated to 78 °C in an oil bath and maintained at that temperature for 4 h. The product was extracted with a separating funnel containing equal volumes of 10% HCl solution and chloroform to isolate the organic phase. The resulting purple crystals had a synthesis yield of 69.6%. The physical data, as determined by FTIR and ^1^H NMR, are consistent with those reported in the literature [60].

#### 4.3.5. Synthesis and Characterization of the Photoactive Copolymers

The P(MAn-alt-OD) and P(MAn-alt-2MB) were functionalized with the ***SP*** photochromic compound through esterification reaction. As a result, purple and yellow products were obtained. The synthesis and characterization of the **SP** were performed as described in the referenced literature [4,60,62,64]. The synthesis yield was 70.6%, and the physical data, determined by FTIR and ^1^H NMR, were consistent with those previously reported [62]. The characterization by ^1^H NMR was carried out, see Figure S1a of the photoactive copolymers P(MAn-alt-OD)-***SP*** and Figure S1b P(MAn-alt-2MB)-***SP***. ^1^H NMR- spectrum (δ ppm, CHCl_3_) of the P(MAn-alt-OD)-***SP*** copolymer shows the following signals at: 8.00 (2H, m, Ha and Hb); 7.20 (1H, t, Hd); 7.10 (1H, d, Hc); 6.90 (2H, m, He and Hf); 6.77 (1H, d, Hh); 6.66 (1H, d, Hi); 5.88 (1H, d, Hg); 3.79 (2H, m, -CH_2_O-); 3.40 (2H, m, -CH_2_N<); 2.80 to 0.4 (48H, m, 3× –CH<, 18× –CH_2_- and 3× –CH_3_), see Figure S1a. The characterization of P(MAn- alt-2MB)-***SP*** shows the following signals: δ (ppm, DMSO-d6): 2.2 to 1.7 (2H, m width, MAn); 1.6 and 0.6 (40H, S wide, >CH-, -CH2-, -CH3). ^1^H NMR- spectrum (δ ppm, CHCl_3_) of P(MAn-alt- 2MB)-***SP***: the spectrum shows the following signals at: 8.01 (2H, m, Ha y Hb); 7.19 (1H, t, Hd); 7.11 (1H, d, Hc); 6.90 (2H, m, He y Hf); 6.75 (1H, d, Hh); 6.67 (1H, d, Hi); 5.89 (1H, d, Hg); 3.77 (2H, m, -CH_2_O-); 3.40 (2H, m, -CH_2_N<); 2.80 a 0.5 (18H, m, 3× –CH< y 5× –CH_3_) (see Figure S1b).

#### 4.3.6. Preparation of Photoactive Hydrogel Films Functionalized with CdTe/ZnS (NPs)

The photoactive copolymer (1 mg) was dissolved in 1 mL of CHCl_3_ to obtain a 1 mg/mL solution. Subsequently, CdTe/ZnS quantum dots (QDs) were incorporated into the solution at different concentrations (1 and 2 wt%). The solution was stirred vigorously for 15 min to ensure coordination of the -**SP** moiety via the nitro groups of the photoactive copolymer to the surface of the core–shell nanoparticles, in the presence of 0.1 mol.% of 2-hydroxy-4′-(2-hydroxyethoxy)-2-methylpropiofenone (Irgacure-2959) as a photoinitiator and the crosslinker agent (MBA). The copolymer solutions were spin-coated onto a glass substrate. Then the films were activated by UV light, exposed to radiation for 5 min on glass slides using a ramped-rotation speed profile: 500 rpm for about 20 s, then 1800 rpm for about 30 s. The ATR-FTIR spectra of the prepared films were recorded.

## Figures and Tables

**Table 1:** Main vibration bands of the photoactive copolymers-CdTe/ZnS (NPs).

Wavenumber cm^−1^	Functional Groups	Type of Vibration
3400.0	-O-H,	stretching hydroxyl groups
2985.0, 2855.0	-C-H, =C-H,	stretching alkane and alkene
1796.5, 1780.3, 1703.3 1727.9 (with ZnS/CdTe)	-C=O	stretching asymmetric and symmetric maleic anhydride (MAn) and ester (–COOR) in the presence of **ZnS/CdTe**
1612.5	-C=C-	Aromatic rings from ***SP*** moiety
1459.7 and 1458.3	-NO_2_	stretching N-O; -N=O, nitro symmetric from ***SP*** moiety
1476.9, 1456.9 1481.3 (with ZnS/CdTe)	-NO_2_	Nitro group asymmetric from **SP** moiety. This band increases its intensity in the presence of **ZnS**
1269	-C-O-C-	Stretching the cyclic ether from the **SP** moiety
785.7, 744.2, 714.0	Aromatic ring	Stretching C-H;
685.0 747.0, 714.0, 668.3 (with CdTe/ZnS)	**CdTe/ZnS**	interaction with nitro groups from **SP** moiety

**Table 2:** Optical Band gap (*Eg*) * before and after irradiation at 365 nm.

Case	$E_{g}$ (eV)		
	Before	After	Reference
Photochromic agent ***SP***	3.00	2.00	[62]
P(MAn-alt-OD)-***SP***	3.73	3.02	[62]
P(MAn-alt-2MB)-***SP***	3.78	3.20	[62]
ZnS(QDs)	3.95	3.95	[59,65]
ZnS/CdTe (QDs)	3.98	4.00	This work
P(MAn-alt-2MB)-***SP***-ZnS (QDs)	2.98	2.93	This work
P(MAn-alt-OD)-***SP***-ZnS (QDs)	3.17	3.10	This work
P(MAn-alt-2MB)-***SP***-CdTe/ZnS (QDs)	2.93	2.89	This work
P(MAn-alt-OD)-***SP***-CdTe/ZnS (QDs)	3.14	3.07	This work

* The Eg was estimated from Tauc plot equation [64].

**Table 3:** Water contact angle (WCA) values the photoactive polymer films.

Systems	Average Contact Angle	Standard Deviation
Glass substrate	50.0	1.39
P(MA-alt-2MB)-***SP***	69.8	0.07
P(MA-alt-2MB)-***SP***-*CdTe/ZnS*	85.7	0.10
P(MA-alt-OD)-***SP***	58.8	0.10
P(MA-alt-OD)-***SP****-CdTe/ZnS*	82.1	0.60

## Data Availability

The original contributions presented in this study are included in the article/Supplementary Material. Further inquiries can be directed to the corresponding authors.

## Supplementary Materials

The following supporting information can be downloaded at: https://www.mdpi.com/article/10.3390/gels12030202/s1, Figure S1: (a) ^1^H-NMR spectrum of P(MAn-*alt*-2MB)-***SP*** and (b) P(MAn-*alt*-OD)-***SP***.
