# Supplementary material for: Optical Sensing Properties of New Innovative Materials: Interaction of Photoactive Copolymers with Fluorescent Nanoparticles to Create Light-Sensitive Hydrogel Films
Source: Gels. 2026 Feb 28;12(3):202. doi: 10.3390/gels12030202 (PMC13025336; doi:10.3390/gels12030202)

Supplementary Material

Figure Legends

Figure S1: a) ^{1}H-NMR spectrum of P(MAn-*alt*-2MB)-***SP*** and b) P(MAn-*alt*-OD)-***SP***.

Figure S1

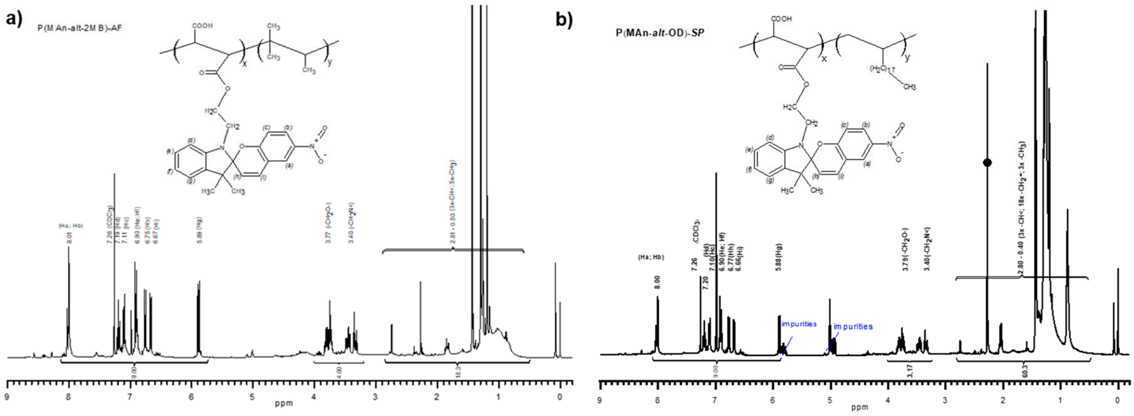

Supplement: Supplementary file 1 [file gels-12-00202-s001.zip › gels-4069758-supplementary.pdf]
